# Impact of Nanoplastics on the Functional Profile of Microalgae Species Used as Food Supplements: Insights from Comparative In Vitro and Ex Vivo Digestion Studies

**DOI:** 10.1021/acs.jafc.4c07368

**Published:** 2024-12-24

**Authors:** Davide Lanzoni, Marisa Sárria Pereira de Passos, Dora Mehn, Sabrina Gioria, António A. Vicente, Carlotta Giromini

**Affiliations:** † Department of Veterinary Medicine and Animal Science (DIVAS), Università degli Studi di Milano, Via dell’Università 6, 29600 Lodi, Italy; ‡ European Commission, Joint Research Centre (JRC), 20127 Ispra, Italy; § CEB − Centre of Biological Engineering, University of Minho, 4710-057 Braga, Portugal; ∥ Institute for Food, Nutrition and Health, University of Reading, Reading RG6 5 EU, U.K.

**Keywords:** Chlorella vulgaris, food supplements, Haematococcus pluvialis, nanoplastics, polyethylene, polystyrene

## Abstract

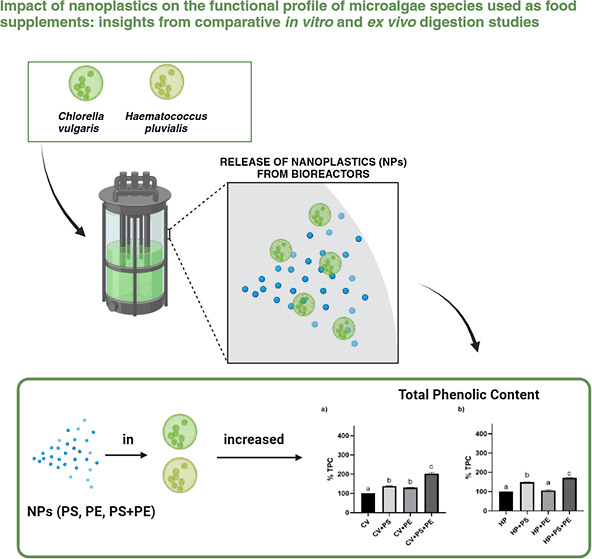

The widespread use of plastics in the food industry raises concerns about plastic migration and health risks. The degradation of primary polymers like polystyrene (PS) and polyethylene (PE) can generate nanoplastics (NPs), increasing food biohazard. This study assessed the impact of PS, PE, and PS + PE NPs on *Chlorella vulgaris* (CV) and *Haematococcus pluvialis* (HP) before and after in vitro and ex vivo digestion, focusing on particle size, polydispersity index, and surface charge. The modulation of total phenolic content (TPC) induced by NP contamination was also evaluated. Results demonstrated that NP behavior varied with the microalgae medium and persisted postdigestion, posing health risks. Significant size increases were noted for PS + PE in the CV and HP. TPC increased significantly with NP exposure, especially PS + PE. These findings underline the need for regulatory measures to ensure food safety in cases of plastic contamination and to address the behavior and toxicity of NPs.

## 1 Introduction

Plastic is a polymer commonly used throughout the world, characterized by versatility, strength, and cost-effectiveness.^1^ In 2020 alone, the global production of plastic products reached nearly 320 million tonnes, a number that is anticipated to increase dramatically by 2050,^2^ with an estimated production of 1.1 billion of new products.^3^ The widespread use of plastic products generates a huge amount of plastic waste, of which only 9% is properly recycled.^4^ Within all uses of plastics, special attention has been given to the food sector, due to safety concerns that are fundamentally associated with the plastic materials propensity to migrate into foodstuffs and, consequently, to their potential human and animal health impact.^4−6^

Among the most used plastic polymers are polystyrene (PS) and polyethylene (PE).^7^ Although plastic polymers are generally recognized as chemically inert materials, microplastics (MP, <5 mm) and nanoplastics (NP, <1000 nm) particles, resulting from degradation of primary plastic products, may however “carry” a masked biohazard.^8^ The small size of MPs and NPs is a critical factor in their interaction with the human organism. Specifically, they can enter the human body through three main routes: skin contact, inhalation, and ingestion.^9^ Of these, the latter deserves particular attention, as it is estimated that the annual intake of plastic particles per person is approximately 39.000–52.000.^10^ Contact between MPs and NPs and the food matrix can occur in multiple ways: (a) environmental contamination; (b) during transport and storage; (c) during food processing; (d) distribution; and (e) food packaging.^11,12^

However, data gaps exist in the understanding of the effects associated with mixtures of plastics on food matrices and the implications of plastic transformations once undergoing gastrointestinal digestion.^13,14^ In accordance with the recommendations of the European Food Safety Authority,^8^ a comprehensive understanding of these dynamics is of high priority, not only because fluids in the digestive tract can change the surface properties of plastics, leading to the formation of a protein corona that can alter the bioavailability of food nutrients but also because of the high specific surface area of NPs, which makes them more easily at risk of toxicity due to the impact of their greater reactivity (given their smaller size) on their fate, more easily translocating cellular barriers, and consequently highly susceptible to being absorbed from the gut.

In a rapidly changing world, food supplements are becoming increasingly relevant to cope with the altering dietary habits, the growing health awareness, and the aging of the global population, as due to their concentrated nutrient content, these play a crucial role in supporting overall health and well-being alongside regular diets.^15^ In this context, microalgae are enjoying great success in the food and feed industry for the production of functional foods.^16,17^*Chlorella vulgaris* (CV) and *Haematococcus pluvialis* (HP) are two microalgae species gaining prominence as food supplements due to their rich nutritional profiles and bioactive compounds. While the first is certainly the most well known due to its important nutritional profile (55–67% protein, 7–15% lipids, and 9–18% dietary fiber on a dry matter basis) and important bioactivity,^18,19^ the last is attracting particular interest due to the production (around 5% of dry matter) of astaxanthin (3,3′-dihydroxy-β, β-1-carotene-4,4′-dione), a potent antioxidant contributing to its wide recognition for antitumor, antiaging, and anti-inflammatory benefits,^20^ being therefore acknowledged by the European Regulation 2015/2283 on novel foods as a food supplement.^21^ Among the functional components, microalgae are recognized for their high phenolic content, an important parameter known to have interesting beneficial effects on human health.

This plays a key role in the food sector, especially if one considers that cultivation conditions can influence the total content.^16,17^

Nevertheless, important data gaps are yet to be addressed for a comprehensive safety profile of microalgae as food supplements; e.g., studies are still needed to understand how plastic mixtures affect the functional profile of microalgae at the different stages of the digestive process. On the other hand, microsize plastic particles, but not NPs, have received extensive research attention regarding their presence in food matrices and potential impacts on food safety and quality. Studies have documented their accumulation in various food items and assessed their interactions with food components, highlighting concerns over contamination and potential health risks.^22^ Adding to this, analytical techniques for detecting and quantifying MPs are well-established,^23^ facilitating a deeper understanding of their behavior and effects in food systems. In contrast, NPs represent a newer area of research with limited available data on their specific impacts on food matrices.

A growing recognition of NPs as a significant human health concern, alongside the increasingly exploration of microalgae as sustainable high-added value sources for food supplements, highlights the need for studies targeting the interactions and transformations of NPs, especially as mixtures of polymers, once in contact with food matrices, not only before but particularly during the digestive process, to more accurately inform the regulatory measures aiming to safeguard food quality and ensure consumer safety regarding plastics use for the food sector.

To date, the study of the toxic effects of plastic contaminants on microalgae has focused on growth inhibition, morphological changes, and modulation of essential pigments.^24^ On the other hand, no studies so far have investigated the impact of NPs on the production of phenols by microalgae. These secondary compounds serve as a defense mechanism for microalgae against biotic and abiotic stresses.^25^ Understanding phenol production is paramount for ensuring the safety and efficacy of microalgae-based food supplements for consumers, as these may influence their bioactivity and potential health benefits. Phenols possess antioxidant properties that can contribute to human health by protecting cells from oxidative damage and inflammation.^25^

Although, to the best of our knowledge, there are no live microalgae in food applications. Live microalgae can be exposed to plastic materials during industrial culture systems where microalgae are commonly cultivated in large-scale plastic bioreactors, especially PE bags or photobioreactors.^26^ During the cultivation phase, contamination by plastic nanoparticles can occur, especially if the plastic material is degraded or infiltrated the culture. This step in the supply chain can lead to the live microalgae being exposed to plastic contaminants prior to processing, creating a potential contamination pathway with plastic nanoparticles, which can be transferred into downstream products even if the algae are subsequently processed into non-living forms.

By testing live microalgae, we are modeling the highest-risk scenario: if there is plastic contamination during the cultivation phase, when the algae are still alive, it is possible to understand and mitigate the likelihood of contamination at later stages of the production process.

Taking the above-mentioned points, in this work, we aimed to understand the effects on the functional profile of two microalgae species (CV and HP) relevant as food supplements upon single (PS, PE) and NP mixture (PS + PE) exposure (the latter poorly investigated in the literature) before and after in vitro and ex vivo digestion. Variations on the microalgae total phenolic content (TPC) were assessed using the Folin–Ciocalteu method. To investigate NP transformations before and after the two digestion approaches, different analytics were considered. More precisely, particle size (hydrodynamic diameter) and polydispersity index (PdI) were determined by dynamic light scattering (DLS), while modifications on NP surface charge were measured by zeta (ζ) potential analysis.

## 2 Materials and Methods

**Figure 1:**
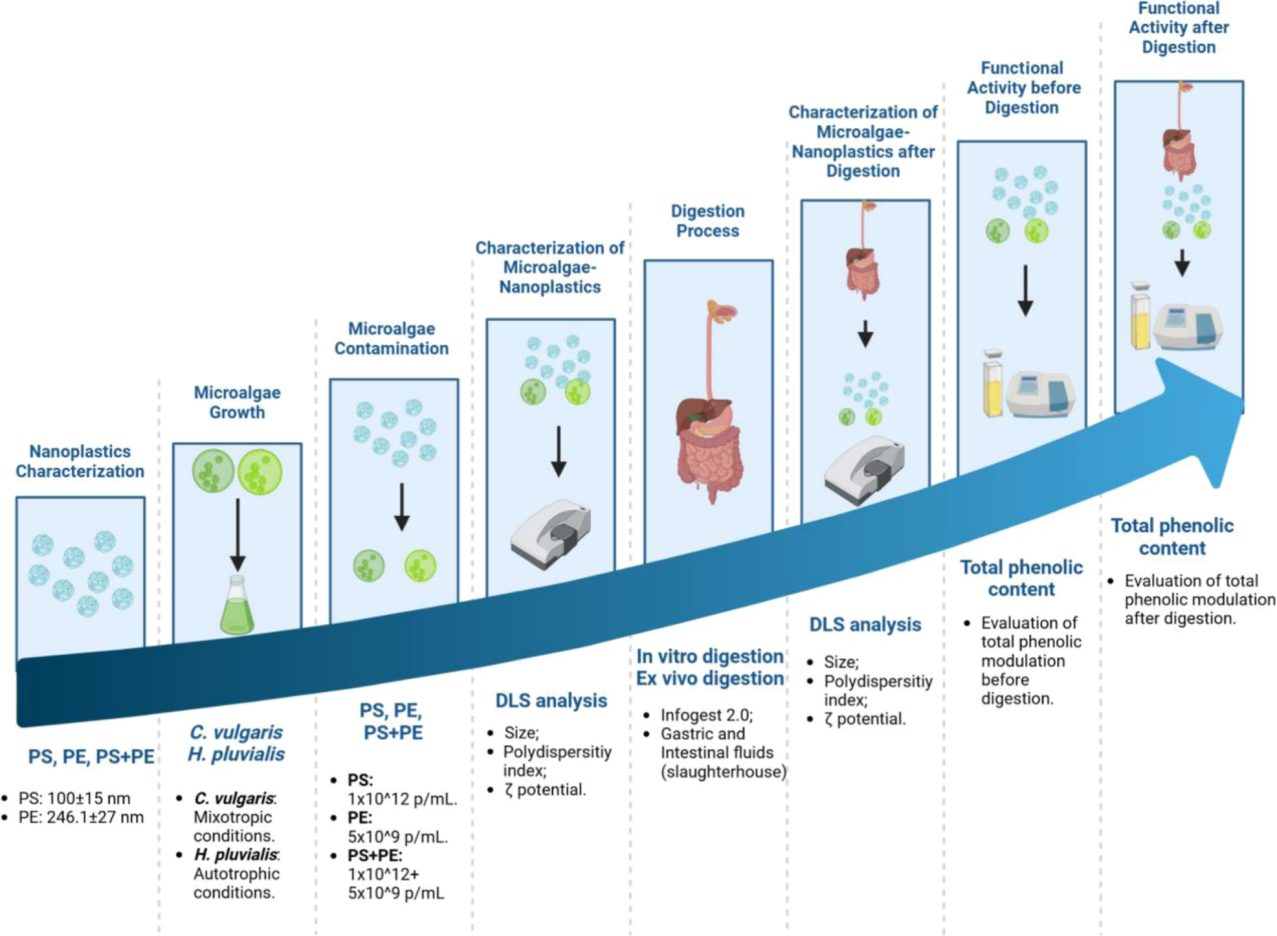
Experimental design. Figure created with BioRender.com.

The experimental design is depicted schematically in Figure 1.

### 2.1 Nanoplastics

Size analytical standard (spherical, monodisperse) plain (not functionalized) PS particles of physical diameter 100.0 (±15) nm (CV, coefficient of variance of 15%) at 2.5% (w/v) solids in aqueous dispersant were acquired from Polysciences (polybead microspheres, 00876-15). Particle average diameters are in agreement with calibration and traceability procedures from the National Institute of Standards and Technology (NIST, USA). PE NPs were in-house synthetized following an oil-in-water emulsion precipitation protocol.^27^ Raman spectroscopy was conducted to characterize the colloidal PE plastic particle chemical composition to ensure that the polymer was not chemically modified or degraded during the synthesis (unpublished data). PE NP size distribution and particle density were determined using a centrifugal sedimentation method (Supporting Information, Figure S1). Gathering data on these particular physical-chemical characteristics is key to providing insights into the behavior of the PE NPs under physiological conditions, particularly during in vitro and ex vivo digestion (as intended). By knowing these parameters, the stability, aggregation tendencies, and potential bioavailability of the PE NPs can be inferred. Factors that are essential to elucidate how particles interact with the food matrices, as these can influence their transformations, transport, and toxicity in the gastrointestinal tract.

### 2.2 Microalgae Growth and Nanoplastics Contamination

Mother strain (axenic) cultures of the two microalgae species used as food matrices for NP exposure experiments were acquired from the BMCC Basque Microalgae Culture Collection: [BMCC127] CV (Trebouxiophyceae) and [BMCC673] HP (Chlorophyceae). Once at controlled (light intensity, photoperiod, temperature, and humidity) laboratory conditions under an environmental chamber (CLR Srl, Z01-S-029), the estuarine (Santurce, Spain) microalgae species (the first) was cultured in mixotrophic conditions using industrial dairy waste (hydrolyzed cheese whey) as organic carbon source,^28^ while the freshwater (Amurrio, Spain) microalgae species (the latter) was cultured in autotrophic conditions using the Blue Green 11 (BG-11) medium, a widely reported mainstream medium for microalgal biomass and lipid production.^29^ To determine the appropriate microalgae cell density to use for the experiments, changes in the number of cells mL^–1^ were monitored in the corresponding growth media overtime by regular sampling and counting of the cells using a Neubauer chamber under an ECLIPSE Ts2 inverted microscope coupled to a DS-Fi3 digital camera. Specific growth curves were then established for the two microalgae species (Supporting Information, Figure S2) that permitted the optimal nominal concentrations (cell density) to be defined for the contact tests and therefore ensured that the cells were at the exponential growth phase during exposure to NPs.

For a constant growth rate at *T*_0_, precultures derived from corresponding microalgae mother strain cultures were prepared 48 h prior the contact tests to NPs in respective growth media. *C. vulgaris* at 3.80 × 10^7^ cells mL^–1^ and HP at 1.75 × 10^7^ cells mL^–1^ were then exposed to 1 × 10^12^ part mL^–1^ of standard PS and 5 × 10^9^ part mL^–1^ of in-house synthesized PE, either as individual (single) exposure or combined (mixture), under stirring at the same environmental chamber as the microalgae mother strain cultures for 24 h.

As plastic nanoparticle size and density significantly drive their behavior and interaction with microalgae cells as food matrices in in vitro and ex vivo digestion, NP test (nominal) concentrations were selected considering the following rationale: (1) PS NPs (100.0 ± 15 nm) have a higher density (1.050 g cm^–3^) and a larger surface area-to-volume ratio corresponding to a greater number of particles per unit mass diffusing faster; however, these are particularly prone to higher sedimentation rates toward the bottom of the exposure vessel, therefore decreasing the possibility of encounters to microalgae cells that are largely suspended in the exposure media—a higher test (nominal) concentration (1 × 10^12^ particles mL^–1^) was therefore set to ensure sufficient exposure, despite the major propensity of aggregation and (or) agglomeration events; (2) as larger particles, PE NPs (246.1 ± 27 nm, Supporting Information, Figure S1) tend to exhibit a different exposure profile due to their lower density (0.882 g cm^–3^, Supporting Information, Figure S1) and smaller surface area-to-volume ratio resulting in fewer particles per unit mass interacting to microalgae cells at lower diffusion rates, but being more likely to remain suspended in the exposure media due to slower settling—therefore, despite the lower (as compared to PS NPs) test (nominal) concentration set (5 × 10^9^ particles mL^–1^), their reduced tendency to rapidly sediment or aggregate and (or) agglomerate can still ensure that an adequate number of particles are accessible for effective encounters to microalgae cells. Ultimately, NP test (nominal) concentrations were set above detection limits of the analytics considered in this study (DLS)^30^ which aims to comprehensively assess how different particle characteristics (as above-mentioned) can impact the functional profile of microalgae as food supplements, as well as understand NP behavior and the various transformations that might occur upon interacting with microalgae as food matrices during a digestive process, thereby providing valuable insights into the potential risks associated with the use of NPs in the food sector.

### 2.3 Dynamic Light Scattering Analysis

Batch mode DLS was used to characterize the size (intensity-weighted mean hydrodynamic diameter, *Z*-average) and PdI of standard PS and PE NPs in different microalgae growth media, pre- and postdigestion. Moreover, given the significant (nanoscale) size difference expected between the particle size populations (modes), batch mode DLS was further considered to resolve multimodal NP size distribution once in a (NPs) binary mixture (pre- and postdigestion studies). To investigate NP postdigestion, changes in size (hydrodynamic diameter), PdI, and size distribution once in a mixture to microalgae cell debris (lysate) were measured. Time-resolved DLS was used to characterize the NP behavior (e.g., particle sedimentation, aggregation, and (or) agglomeration events) in the different microalgae growth media during the same exposure time (24 h) as the contact tests (predigestion).

Electrophoretic DLS Mode was used to measure predigestion only (residual enzymes or byproducts of postdigestion can modify the electrical properties of the sample, as interfering on NP particles interferes with the measurement process, influencing the time required for stabilization and the overall accuracy of the results.), the NP electrophoretic mobility in the different microalgae growth media, with and without microalgae cells, for ζ potential assessment and particle surface charge analysis. DLS measurements were conducted using a Zetasizer Nano ZS (Malvern Instruments Ltd., Malvern, UK) at 25 ± 1 °C for a backscattering angle of 173°. Nanoplastics hydrodynamic diameters (*Z*-average) and the dispersity from cumulative analysis were determined according to ISO 22412:2017.^31^ ζ-Potential analysis for NP surface charge assessment was conducted in agreement with ISO 13099-1:2012.^32^

### 2.4 In Vitro Digestion

In vitro digestion studies were performed following the standard static INFOGEST.^33,34^ The reagents and enzymes used were purchased from Sigma Chemical Co. (St. Louis, MO, USA). Simulated digestion fluids for oral (SOF), gastric (SGF), and intestinal (SIF) phases were prepared in agreement with Brodkorb et al. (2019) and Minekus et al. (2014).^33,34^ For the oral digestion phase, microalgae cells precontaminated (for 24 h) to NPs were diluted to the final (nominal) cell density of 9.5 × 10^6^ cells mL^–1^ and 4.4 × 10^6^ cells mL^–1^, respectively, for CV and HP in SOF, and incubated for 2 min at 37 °C to α-amylase (75 U mL^–1^, pH 7.0) under stirring. Subsequently, the oral bolus was diluted with SGF and pepsin (2000 U mL^–1^, pH 3.0) and incubated at 37 °C for 2 h under stirring. At the end, the gastric chyme was diluted with SIF and incubated with bile salts (10 mM, pH 7.0) and the pancreatic enzymes (100 U mL^–1^, pH 7.0) for 2 h at 37 °C, under stirring. Once the digestion process was completed, the samples were centrifuged following the protocol of Gonçalves et al. (2021),^35^ with the aim of separating the digested fraction (supernatant) from the undigested fraction (pellet). Subsequently, the samples were stored at −20 °C until further analysis to characterize and investigate changes on the NP primary features and how these affect their interactions with the microalgae as food matrices, the behavior modifications that can occur once these are in contact with the digestive fluids, and discuss whether these can impact nutrients bioavailability and absorption upon the digestive process, but also to provide insight into the NP-exposure-associated variations on the microalgae TPC that can affect their nutritional value as food matrices.

### 2.5 Ex Vivo Digestion

The ex vivo digestion process represents an innovative and, as yet, unexplored method in the literature for replicating digestion conditions. Ex vivo digestion was performed using gastric and intestinal fluids collected from slaughter-housed (Lodi, Italy) pigs (*n* = 24), aged between 50 and 110 days, as reported by Lanzoni et al. (2024).^17^ Microalgae cells precontaminated (for 24 h) with NPs were diluted in the gastric and intestinal pig fluids to the final (nominal) cell density of 9.5 × 10^6^ cells mL^–1^ and 4.4 × 10^6^ cells mL^–1^, respectively, for CV and HP. At the end of the digestion process, samples were centrifuged at 18.700 RCF for 30 min at RT, thus separating the digested fraction (supernatant) from the undigested fraction (pellet).^35^ The fractions obtained were frozen at −20 °C until further analysis, as above-mentioned.

### 2.6 Total Phenolic Content

For quantification of microalgae TPC, the protocol reported by Attard (2013)^36^ was performed with minor modifications.^25^ The reagents used (tannic acid, Folin–Ciocalteu and sodium carbonate) were purchased from Sigma Chemical Co. (St. Louis, MO, USA). Briefly, 0.100 mL of each sample was incubated to 0.500 mL of Folin–Ciocalteu (diluted 1:10 with distilled H_2_O) and 0.400 mL of sodium carbonate (10.589 g in 100 mL of distilled H_2_O) for 20 min in the dark at RT. At the end of the incubation period, the absorbance of the resulting blue color was measured on the samples using a spectrophotometer at λ = 630 nm. Appropriate blanks were included in the analysis. Values for TPC (expressed in %) were normalized toward the control (microalgae only).

### 2.7 Statical Analysis

The size (*Z*-average, nm), PdI, and ζ-potential (mV) of the NPs and the TPC of the microalgae pre- and postdigestion (in vitro and ex vivo) was analyzed using a one-way ANOVA followed by Tukey’s multiple comparison using GraphPad Prism (9) 9.3.1 (GraphPad Software Inc., San Diego, CA, USA). All parametric assumptions were met. Data was expressed as the average (arithmetic mean) ± SEM of at least three independent experiments. Values were considered statistically significant for a 95% confidence interval (*P* value = 0.05).

## 3 Results and Discussion

### 3.1 Predigestion Characterization of Nanoplastics

To better understand the biological impact of NPs, in addition to the nominal values, it is necessary to perform an in-depth characterization of their physicochemical properties in the exposure media.^37^ As accordingly, the size (*Z*-average), PdI, and ζ-potential of PS, PE, and PS + PE in the different microalgae culture media was investigated (Table 1).

**Table 1:** Size (*Z*-Average), PdI, and ζ-potential of PS, PE, and PS + PE Nanoplastics in Microalgae Growth Media^a^^,^^b^

sample	*Z*-average (d nm)	PdI	ζ-potential (mV)
PS in CV growth medium	85.32 ± 0.32^a^	0.05 ± 0.01^a^	–10.60 ± 0.26^a^, pH 7.65
PS in HP growth medium	87.86 ± 0.09^a^	0.10 ± 0.01^a^	–11.93 ± 1.09^a^, pH 7.36
PE in CV growth medium	207.50 ± 1.18^b^	0.13 ± 0.01^b^	–15.57 ± 0.29^b^, pH 7.68
PE in HP growth medium	205.30 ± 1.45^b^	0.13 ± 0.00^b^	–24.60 ± 0.95^c^, pH 7.17
PS + PE in CV growth medium	267.20 ± 4.84^c^	0.33 ± 0.02^c^	–19.03 ± 0.17^b^, pH 7.73
PS + PE in HP growth medium	204.67 ± 1.77^b^	0.17 ± 0.00^b^	–17.23 ± 0.98^b^, pH 7.13

a Different superscript letters indicate statistically significant differences among groups (*P* < 0.05). Results are reported as mean ± SEM.

b PdI = polydispersity index; PS = polystyrene; PE = polyethylene; CV = *C. vulgaris*; HP = *H. pluvialis*. CV growth media (only, no NPs) pH = 7.69 ± 0.03. HP growth media (only, no NPs) pH = 7.22 ± 0.10.

**Figure 2:**
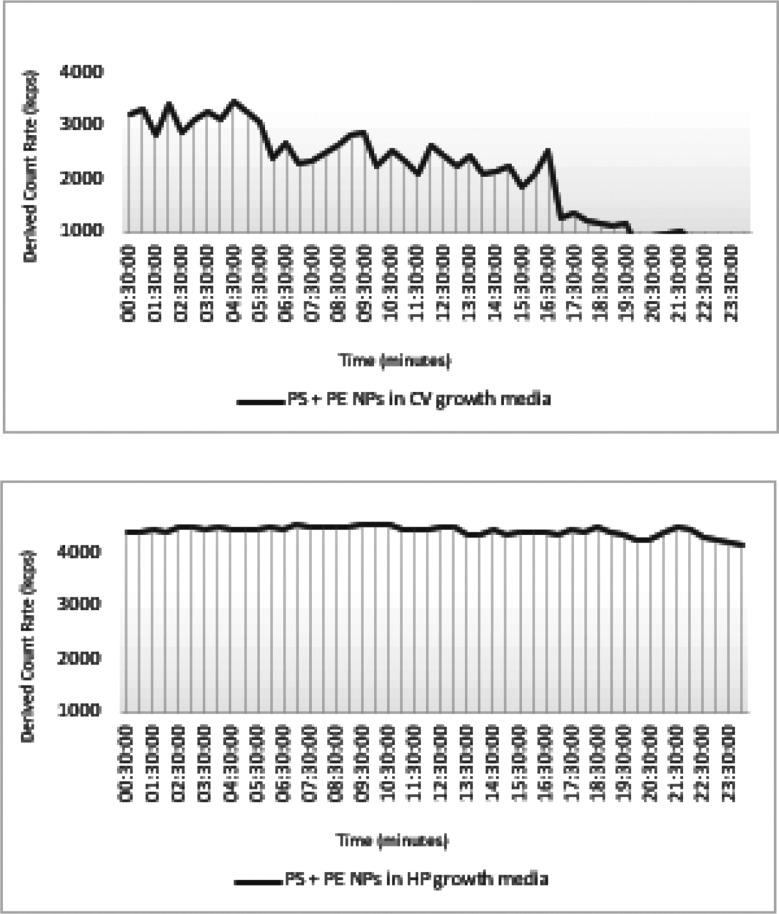
Derived count rate of PS + PE nanoplastics (NPs) in microalgae growth media overtime. PS = polystyrene; PE = polyethylene; NPs = nanoplastics; CV = *C. vulgaris*; HP = *H. pluvialis*; and Kcps = kilo counts per second.

All these parameters are crucial in determining the colloidal stability of NPs, which in turn influences their reactivity.^38,39^ Although PS NPs showed a lower intensity-weighted mean hydrodynamic diameter in both CV (85.32 ± 0.32 nm) and HP (87.86 ± 0.09 nm) growth media, these values are still within the range of the expected standard particle nominal size (100 ± 15 nm). Similarly, PE NPs *Z*-average did not differ from the expected determined particle nominal size (Supporting Information, Figure S1), as also no differences in intensity-weighted mean hydrodynamic diameters were recorded among CV (207.50 ± 1.18 nm) and HP (205.30 ± 1.45 nm) growth media. Once in a binary mixture (PS + PE), though, different *Z*-average results were recorded in the two microalgae growth media. While in HP growth medium, PS + PE showed an intensity-weighted mean hydrodynamic diameter (204.67 ± 1.77 nm) highly comparable to single PE (independently of the microalgae growth media), in CV growth medium a significantly higher *Z*-average (*P* < 0.05) was recorded (267.20 ± 4.84 nm). By DLS analysis, the intensity of the light scattered by a particle is proportional to the sixth power of its radius (Rayleigh scattering theory); therefore, larger particles are detected more readily than smaller ones.^40^ Larger particles also diffuse more slowly due to Brownian (random) motion that permits easier-to-measure fluctuations in the scattered light, leading to a higher signal-to-noise ratio and making the detection of larger particles more prominent.^41^ It is therefore understandable that in the NP binary mixture, the data analysis algorithms used in DLS could not deconvolute the signal of PS (smaller particles) over the strong scattering of PE (larger particles) that dominates, overshadowing and masking the detection of the (weaker) signal of PS. It was further interesting to notice that aside from the higher *Z*-average values obtained for PS + PE in the CV growth media, a higher PdI value (0.33 ± 0.02) was detected, suggesting the occurrence of agglomeration and (or) aggregation events, as in accordance with Seoane et al. (2019),^42^ who demonstrated PdI values greater than 0.20 to represent an agglomeration and (or) aggregation factor among NPs. Additionally, once investigating the count rate overtime (Figure 2), it could be observed that not only the T_0_ count rate of the NP binary mixture in the HP growth media was higher (∼4000 kcps) as compared to the one in CV growth media (∼3000 kcps) but also constant, remaining stable along the measurement time (24 h). On the contrary, an erratic count rate was recorded overtime for the PS + PE NPs in CV growth media, showing a significant decrease in the number of particles in the detection volume (count rate ∼2000 kcps) for the first 12 h (Figure 2), therefore corroborating the hypothesis of particles clustering overtime, becoming larger than the expected nominal size and being more prone to settle out, thus lowering the count rate because of the fewer particles contributing to the scattering process.

ζ-Potential is key to understanding the stability of colloidal dispersions, influencing cell permeability, protein interactions, and toxicity.^43^ It measures the magnitude of the electrostatic forces at the slipping plane of a particle or the degree of repulsion among adjacent and similarly charged particles in a colloidal system. ζ-Potential threshold values of absolute 30 (negative or positive) mV are often cited as the boundaries for colloidal stability.^37,39,44^ When having a ζ-potential outside this range, the repulsive forces among particles are higher, making these less prone to agglomerate and (or) aggregate, thus being considered more stable. As shown in Table 1, the ζ-potential values are outside the boundaries for colloidal stability for the NPs tested. PS NPs showed similar values of ζ-potential in CV (−10.60 ± 0.26 mV) and in HP (−11.93 ± 1.09 mV) growth media, but for PE NPs, a more negative charge close to −30 mV was recorded in HP growth media (−24.60 ± 0.95 mV), therefore suggesting that particle stability is higher than in CV growth media, for which a ζ-potential similar to PS NPs was detected (Table 1). In agreement, it was also in HP growth media that once in a binary mixture, the NPs showed less propensity to agglomerate and (or) aggregate overtime (Figure 2).

**Figure 3:**
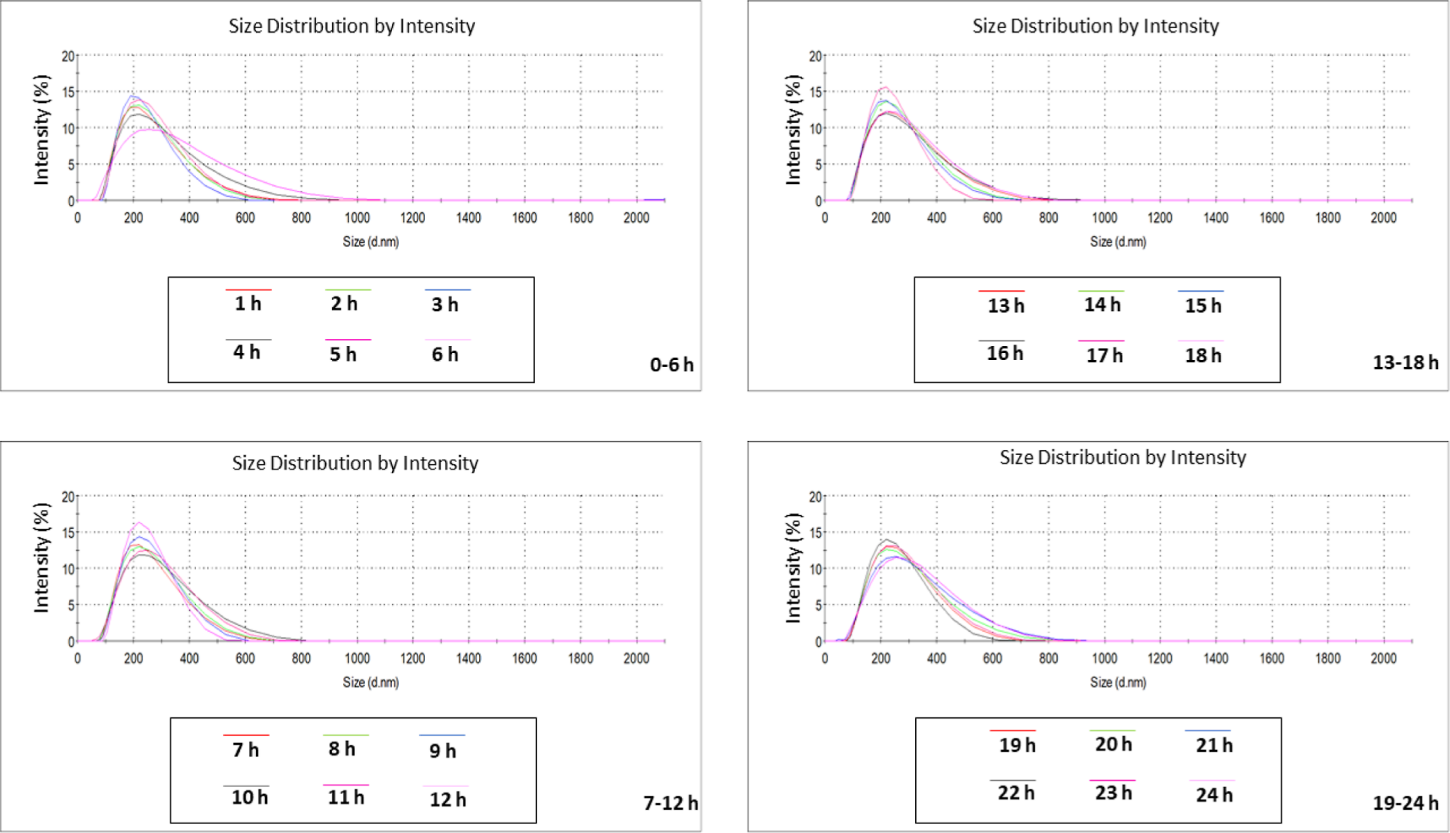
Analytical characterization of the size distribution of PS + PE NPs in *H. pluvialis* growth media for 24 h. Results are expressed in d nm and reported every 6 h.

**Figure 4:**
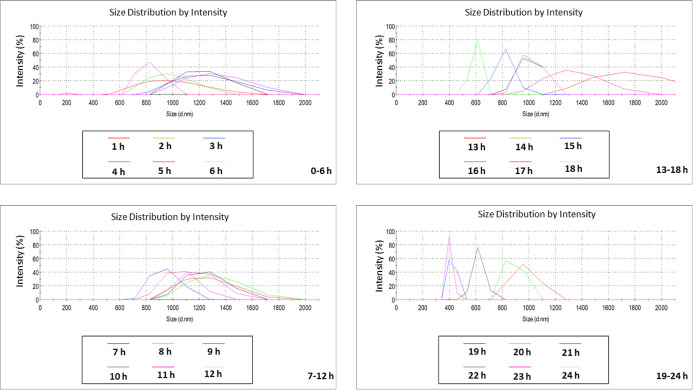
Analytical characterization of the size distribution of PS + PE NPs in *C. vulgaris* media for 24 h. Results are expressed in d nm and reported every 6 h.

Given the different behavior of PS + PE NPs in the two microalgae growth media, their stability overtime was investigated using DLS analysis to collect real-time data on NP size distribution and monitor the changes on particles size that might suggest agglomeration and (or) aggregation events. Measurements were conducted for 24 consecutive h to simulate the same exposure time of the microalgae precontamination tests, as reported in Figures 3 and 4.

Size distribution results of PS + PE NPs in the HP growth medium (Figure 3) corroborated the previously anticipated stability. More precisely, along the 24 h analysis, DLS recorded identical (overlapping) peaks showing a maximum intensity (%) at ∼200 nm, confirming not only that PE NPs indeed mask the scattering signal of PS NPs but also permitting to pre-empt that no agglomeration and (or) aggregation events will tend to occur during the microalgae precontamination contact tests. In contrast, an inconsistent and reversible overlapping of the intensity peaks overtime was recorded for PS + PE NPs in the CV growth medium (Figure 4), demonstrating that the particles tend to highly agglomerate rather than aggregate since a wider size distribution can be observed with intensity peaks that might suggest the presence of both single particles and loosely bound clusters, indicating therefore a highly heterogeneous mixture as according to the increased (>0.20) PdI values previously detected (Table 1). Indeed, distinct from aggregates, agglomerates are formed by two or more particles held together by weak physical–chemical interactions in a reversible process.^45^ This reversibility is clearly noticeable in Figure 4, with the alternation of random major and minor intensity peaks recorded every h. The above-mentioned differences are, to some extent, probably due to the distinct salt composition of the two microalgae growth media that can influence their ionic strength and the electrostatic interactions among NPs. However, several other factors can influence the agglomeration of the NPs. Among these, pH and the presence of additives and dispersants are particularly relevant.^46,47^ In fact, sodium cholate was used in PE NP in-house synthesis as an anionic surfactant for particle steric stabilization. It can adsorb onto the surface of PE NPs, imparting a significantly negative surface charge, as actually it was recorded in HP growth media that is mostly neutral (Table 1). At this pH, due to the deprotonation of surface groups or the adsorption of ions from the HP growth medium, PS NPs are just slightly negatively charged (Table 1). ζ-Potential results suggest that once in a binary mixture, while PE NPs might contribute moderately to stabilize the (closed) system, the PS NPs’ tendency toward instability could still trigger aggregation and (or) agglomeration events. However, the (estimated) ionic strength of HP growth media at neutral pH is relatively low (∼0.03 M). A thicker electrical double layer can then be formed around the particles that imposes important electrostatic repulsion forces, therefore leading to greater stability (Figure 3). Inversely, at a slightly basic pH, the higher ionic strength estimated for CV growth media (∼0.15 M), can compress the electrical double layer around the particles, reducing the range of electrostatic repulsion, thus permitting these to approximate enough to agglomerate, as it seemed to occur (Figure 4).

Knowledge gathered on the behavior of PS and PE NPs in a closed system is key to understanding their dynamics once in an open system, as it is the case of the human gastrointestinal tract and the associated digestive process. Upon ingestion of food supplements, such as microalgae, contaminated with NPs that migrated from the (plastic) bioreactors or package, particles that come into contact with biological fluids (saliva, gastric juice, or intestinal fluids) encounter a variety of biomolecules (proteins, lipids, and enzymes) that can adsorb onto their surface, forming a “corona” coating. As a result, heteroagglomerates (combination of polymer types or bonds with other naturally occurring particles) can then occur that cause changes on NP density, impacting their buoyancy and propensity to deposit,^48^ which ultimately can modify the bioavailability of nutrients derived from digested food.

### 3.2 Interaction of Nanoplastics and Microalgae on Pre- and Post-in Vitro and Ex Vivo Digestions

**Figure 5:**
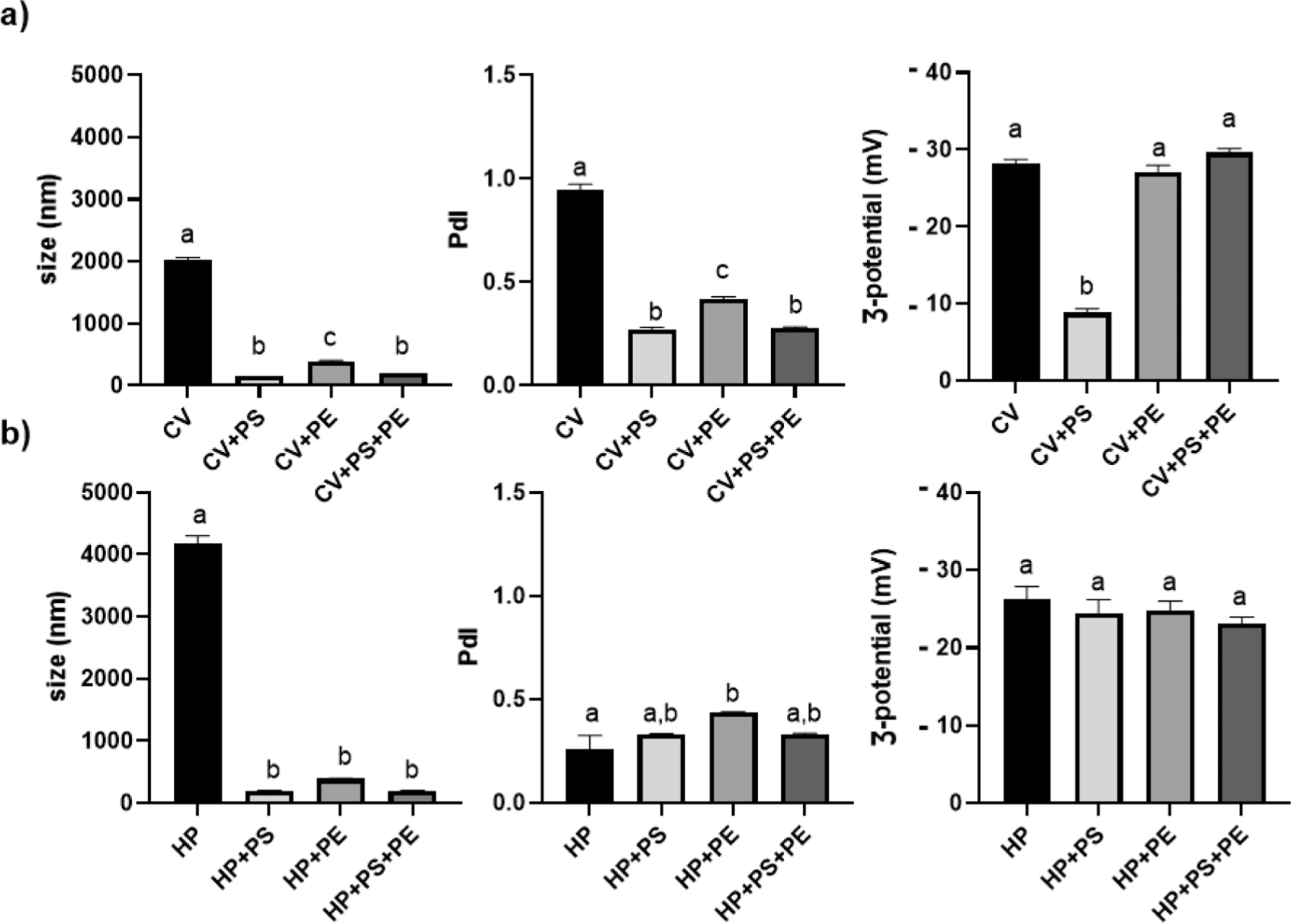
Size (*Z*-average) (nm), polydispersity index (PdI), and ζ-potential (mV) of *C. vulgaris* (a) and *H. pluvialis* (b) before digestion. Different superscript letters indicate statistically significant differences among groups (*P* < 0.05). PS = polystyrene; PE = polyethylene; and CV = *C. vulgaris*; HP = *H. pluvialis*.

Prior to digestion studies, microalgae cells of the two species were contaminated to NPs on a contact test. For a better understanding with the multiplicity of interactions that can occur among NPs and the food biomatrix, a predigestion analytical characterization of the microalgae suspensions of NPs was conducted (Figure 5).

Polydispersity index values of PS, PE, and PS + PE increased in both microalgae growth media (Figure 5), as compared to those observed in the absence of microalgae cells (Table 1). At the same time, although minimal changes were reported for the ζ-potential, values were still outside of the boundaries for colloidal stability, as previously referred. Moreover, the size (*Z*-average) of the NPs was significantly affected but still easily distinguishable from those measured for the microalgae (only) cell suspensions (Figure 5). More precisely, a size of 2040 ± 27.43 nm was recorded for CV, confirming the values reported in the literature.^49^ For HP, the cell size range was 4169 ± 133.67 nm. As documented in the literature, HP can reach 30 μm, although this value is dependent on the replicative stage.^50^ Specifically, this size can be reached during the hematocyst phase, also known as the ’red nonmotile astaxanthin accumulated encysted phase’, the last step of the life cycle.^51^ In the case of our study, the smaller size observed is correlated to the proliferation (exponential) phase (green vegetative palmella), the phase selected for the microalgae contact tests with NPs. As can be observed in Figure 5, the intensity-weighted mean hydrodynamic diameter of single NPs, but not in mixture, showed a significant increase once in coculture with the microalgae cells. For PS NPs in coculture to CV, a smaller value was recorded (146.17 ± 0.32 nm) than that obtained in coculture to HP (197.30 ± 5.18 nm). A trend that was not observed though for PE NPs, for which no relevant particle size differences were recorded between the microalgae cocultures (Figure 5). Interaction among PS NPs and the negatively charged surface of CV cells might not be strong enough to overcome the hydrophobic repulsion forces, resulting in the less pronounced agglomeration as compared to PE NPs. At neutral pH, despite electrostatic interactions being weaker, repulsion forces prevent larger-scale aggregation but are not enough to impede the formation of stable two-particle agglomerates (dimers). An outcome that can be due to a combination of reduced electrostatic repulsion, dynamic equilibrium in the system that prevents larger aggregate formation, and specific molecular interactions facilitated by microalgae exudates. In fact, both CV and HP are microalgae species known to produce extracellular polymeric substances, which are natural biopolymers secreted in response to stress that act as a protective layer against external agents.^52^ Extracellular polymeric substances consist of polysaccharides, enzymes, and structural proteins, among other biomolecules, which, once released to the intracellular media, can coat the NP surface, leading to the formation of a biocorona that consequently can influence their physical–chemical properties (as affecting particle size and density), diffusion, sedimentation, aging, and propensity to cellular membrane translocation or other target interactions.^52,53^

For PS + PE in coculture to CV, PS particles may preferentially adhere to PE NPs or be sterically hindered by these, leading to a size distribution that stabilizes around the size of the larger particles (PE) rather than interacting with microalgae exudates. In fact, the size and surface curvature of NPs are critical factors in determining their affinity toward microalgae extracellular polymeric substances.^54^ For this reason, it is plausible to assume that an increased curvature due to PS + PE NPs preferable agglomeration diminished the contact area and interaction strength between NPs and the biomolecules, reducing but not entirely preventing biocorona formation.^55^ Moreover, the formation of biocorona can counteract ionic effects and stabilize NPs, thus reducing agglomeration through steric interactions, explaining the lower values recorded.^43^ A similar result was obtained for PS + PE in coculture to HP (199.20 ± 2.81 nm). In neutral microalgae growth media, weaker electrostatic interactions lead to less aggregation overall. PE NPs being larger again dominate the size distribution, stabilizing the mixture around their weighted mean hydrodynamic diameter (Figure 5).

**Figure 6:**
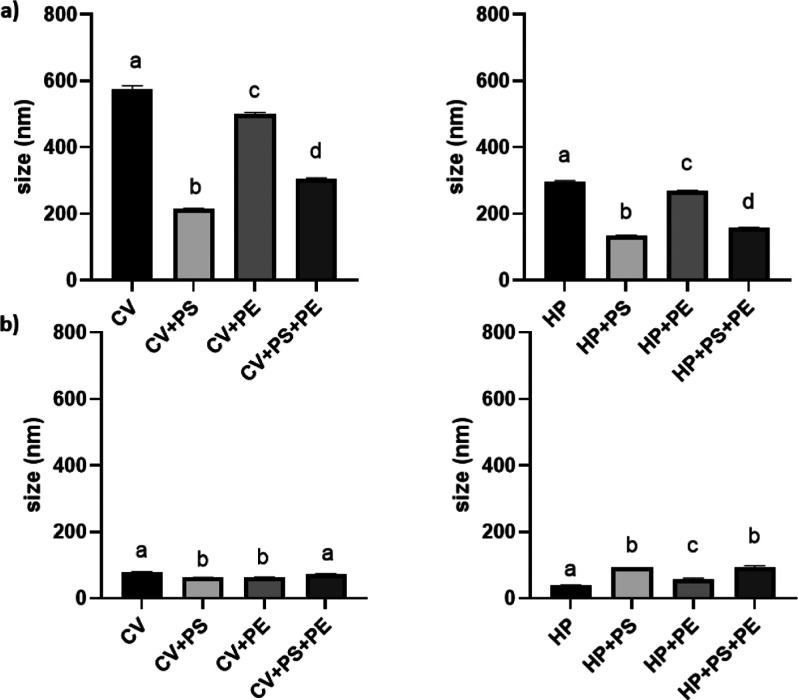
Size (*Z*-average) detected for microalgae suspensions of NPs post (a) in vitro and (b) ex vivo digestion. Different superscript letters indicate statistically significant differences among groups (*P* < 0.05). PS = polystyrene; PE = polyethylene; CV = *C. vulgaris*; and HP = *H. pluvialis*.

**Figure 7:**
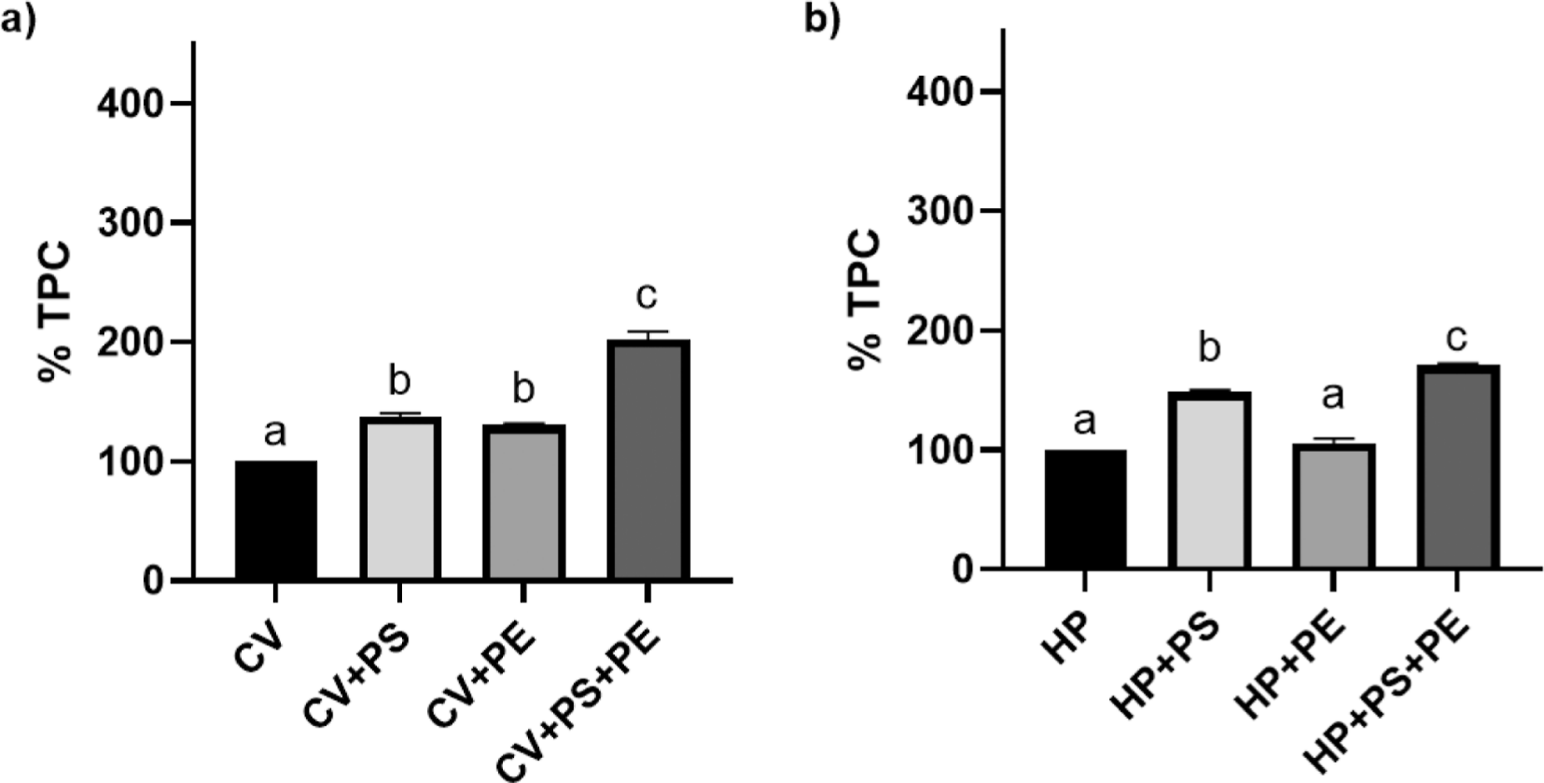
Predigestion analysis of microalgae TPC: (a) *C. vulgaris*; (b) *H. pluvialis*. Data expressed in %, are reported as mean ± standard error of the mean (SEM) (*n* = 3) and are standardized toward the experimental control group (microalgae in growth media only; no NP coexposure). Different superscript letters indicate statistically significant differences among groups (*P* < 0.05). PS = polystyrene; PE = polyethylene; CV = *C. vulgaris*; and HP = *H. pluvialis*.

**Figure 8:**
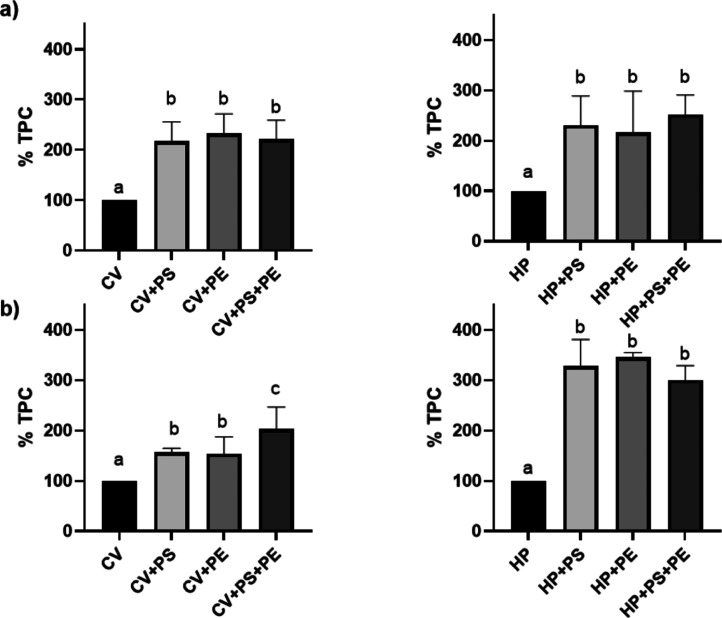
Postdigestion analysis of microalgae TPC: (a) in vitro; (b) ex vivo. Data expressed in %, are reported as mean ± SEM (*n* = 3), and are standardized toward experimental control group (microalgae in growth media only; no NP coexposure). Different superscript letters indicate statistically significant differences among groups (*P* < 0.05). PS = polystyrene; PE = polyethylene; CV = *C. vulgaris*; and HP = *H. pluvialis*.

Given the complexity of the NP-associated interactions with microalgae, as discussed, changes on the NP size (Figure 6) and on the microalgae functional profile (Figures 7 and 8) were investigated in pre- and posthuman digestion simulations to anticipate the scenarios that might occur during cultivation of microalgae in bioreactors.

Obtained results (Figure 6) suggest the ability of NPs to persist in the digested fraction, making thus these available for absorption by intestinal cells, corroborating data as reported by Paul et al. (2024).^56^ Once digested, NPs can: (I) remain in the intestinal lumen, causing local tissue irritation; (II) be absorbed by intestinal cells and released into the lumen following cell death (approximately 72 h later); (III) cross the intestinal epithelium by paracellular pathways (through tight junctions), by per-sorption (that is, through intracellular spaces), or by cells of the intestinal epithelium, therefore reaching the basal side.^56,57^ However, these outcomes are strongly influenced by digestion. In fact, digested NPs are particularly more prone to be absorbed due to the presence of organic matter that facilitates translocation to the intestinal epithelium.^56^ For this reason, it is of great relevance to investigate the individual changes that might occur on NP characteristics and behavior during digestion.

Post in vitro digestion, the size of the microalgae cell debris allows easy distinction from PS, PE, and PS + PE NPs (Figure 6). Interestingly, the size detected for cell debris related to HP (296.77 ± 2.48 nm) was smaller than for those related to CV (574.83 ± 11.04 nm), although, as previously reported, the first was characterized by a larger diameter of the cells (Figure 5). These differences are most probably due to the structure of the cell walls of these microalgae. More precisely, although at the beginning of the growth phase, CV is distinguished by a single microfibrillar layer, the cells rapidly develop a three-layer structure, with a very thick outer layer and a thinner one forming the daughter cell wall.^58^ For this reason, CV cell walls are often classified into a soluble and a rigid fraction, the latter consisting of complex resistant biopolymers and therefore more sensitive to the action of “harsher” enzymes (e.g., chitinases) that are not present in the digestion protocol used.^58^

At the same time, although HP in the palmella stage (the one considered for NP contact testing) is characterized by a complex structure of the cell wall that presents a double membrane layer, one of which is thick and gelatinous, only in the final stage (nonmotile red astaxanthin-accumulating incyst phase) do these cells increase dramatically in volume, becoming surrounded by three thick and tough resistant layers that are difficult to degrade.^59,60^

Furthermore, HP cell walls are sensitive to treatment with hydrochloric acid, which is highly present during the gastric phase of the digestion protocol used.^61^

In regard to NP size characterization post in vitro digestion, while in coculture to HP, NPs showed a reduced weighted mean hydrodynamic diameter (Figure 6) as compared to predigestion data (Figure 5), in coculture to CV, a modest increase of the NP size was recorded. This trend was also confirmed for PS + PE in CV and HP medium with an incremented diameter of 303.53 ± 4.33 and 262.0 ± 0.89 nm, respectively. Biocorona therefore reduce agglomeration through steric interactions, explaining the lower values recorded as compared to NPs in coculture to CV as most likely fewer and larger cell debris were produced as the final product. Obtained results are partially confirmed by the literature, but unique comparisons are difficult due to the numerous factors varying among studies. Krasucka et al. (2022)^62^ reported that despite PS NPs showing no change in primary features, PE NPs were particularly distinguished by a rough and heterogeneous appearance showing deep surface cracks, suggesting a propensity to partial degradation for this plastic polymer.

In another study, Paul et al. (2024)^56^ demonstrated that organic residues derived from microalgae digestion are involved in the increase of NP size, confirming that the higher occurrence of microalgae cell debris can indeed influence the NP propensity to agglomerate, as further corroborated by Li et al. (2023).^63^ In support of this, Fournier et al. (2021)^14^ emphasized the importance of considering the formation of a biocorona on the surface of the NPs due to the adsorption of organic matter as proteins, carbohydrates, and lipids released as a result of the digestive process.

Post ex vivo digestion seemed to result in a more pronounced digestion of the biomatrix, as weighted mean hydrodynamic diameters of 76.89 ± 3.12 and 38.74 ± 1.23 nm were detected for CV and HP, respectively, besides confirming the structural cell wall differences among the two microalgae species. A significantly higher number of smaller microalgae cell debris is therefore expected to interact with the NP surface for ex vivo than for in vitro digestion, increasing the repealing forces among particles due to the formation of an eventually thicker biocorona on the NP surface. These differences were also confirmed by PdI analysis, as in the in vitro digestion, the PdI of the NPs were just slightly modified compared to pre-digestion values (for PS NPs, PdI values of 0.34 ± 0.02 and 0.20 ± 0.01; for PE NPs, PdI values of 0.42 ± 0.02 and 0.40 ± 0.01; for PS + PE NPs, PdI values of 0.45 ± 0.01 and 0.29 ± 0.01 were recorded in coculture to CV and HP, respectively), while ex vivo, the PdI values reported were >0.90, indicating a more polydisperse final digested product due to the more competent digestion of the gastric fluids.

Although there are studies in the literature analyzing the behavior of NPs digested using in vitro systems, to our knowledge, this is the first one using gastric and intestinal juices of animal origin. For this reason, a direct comparison is also difficult due to the nature of the fluids used.

### 3.3 TPC of Microalgae before and after In Vitro and Ex Vivo Digestion

Overall, precontamination of microalgae to NPs significantly increased the TPC (Figure 7). Specifically, exposure of CV to PS NPs (137.86 ± 2.44%) and PE NPs (130.43 ± 1.29%) resulted in a significant (*P* < 0.05) higher phenol content compared to the control group, with no differences being recorded though among the two NPs. However, once exposed to PS + PE NPs (202.10 ± 6.97%), a significantly (*P* < 0.05) higher TPC was recorded than that obtained once CV was exposed to the single NPs. The same trend was registered for HP, although contamination to PE NPs led to a statistically lower TPC (104.72 ± 5.13%) than that recorded in coculture to PS NPs (148.22 ± 2.14%), and highly comparable to the control group. Similarly to CV, also for the coculture of HP to PS + PE NPs, the highest TPC value was recorded (170.84 ± 1.53%). To the best of our knowledge, microalgae phenolic content assessment following contamination with plastic polymers has not been highly investigated in the literature. The majority of the studies focus on the effect of MPs and NPs in modulating growth rates, morphology, chlorophyll content, and photosynthesis processes in microalgae.^23,64^ This trend was confirmed by Menicagli et al. (2022).^65^ In particular, the authors observed how NPs led to an increase in phenols in the shoots of *Cymodocea nodosa*, a seagrass highly described in the literature.^65^

Post in vitro and ex vivo digestions, interesting results were obtained for microalgae TPC (Figure 8).

Tarko et al. (2013)^66^ demonstrated that the absorption and metabolism of phenols in the digestive tract are responsible for their biological properties. Phenolic compound behavior during simulated digestion is adequately described in the literature.^66−68^ More precisely, it is estimated that about 48% of phenols are digested in the small intestine, 42% in the large intestine, while only a small part corresponding to ca. 10% remains bound to the source matrix.^66^ As confirmed by Ginsburg et al. (2012),^67^ although the partial digestion of phenols begins in the oral cavity, it is only in the gastric cavity that the action of the acid pH allows their release.^17,68^ The reported release of phenols following the digestive process would also explain the higher TPC values obtained, compared to those observed in predigestion, although the instability of phenols in an alkaline environment, such as that typical of the small intestine, and in particular, during pancreatic action, leads to the transformation of these compounds into unknown secondary structures with different bioactivity and bioaccessibility.^69^

In the case of microalgae, the production of TPC is most likely associated with a NP-induced stress response; however, it is difficult to ascertain with the current data whether phenols released as a result of the digestive process play a beneficial role in animal and human health. As reported by Halliwell (2008),^70^ no in vivo data are available in the literature on the ability of phenols to act as antioxidants or pro-oxidants in the stomach, intestine, and colon, sites where these may be present at higher concentrations in the organism. However, no evidence of systemic pro-oxidant effects by phenolic compounds has yet emerged after absorption.^70^

In light of the above, although the high phenol content in the treated microalgae suggests that CV and HP are able to cope with the restrictive conditions induced by NPs, the effect of these compounds on animal and human health needs to be further investigated.

In conclusion, the obtained results permitted to demonstrate that NP behavior and the various transformations occurring whether as single particles on in a binary mixture or upon interacting with microalgae as food matrices (predigestion) were highly influenced by the microalgae growth media. Postdigestion studies revealed that NPs were indeed detected in the digested fraction, indicating a potential risk to human and animal intestinal health. Finally, the increase recorded in the phenolic content in NPs precontaminated microalgae used as food matrices upon in vitro and ex vivo digestion suggests a complex interplay between the polymer particles and the biological components (microalgae cells and cells debris, fluids, enzymes, and other biomolecules derived from the digestive process). While enhanced phenolic content could offer some antioxidant benefits, the presence of NPs in the digested fraction might introduce higher potential risks that need to be thoroughly investigated. Understanding these dynamics is key to understanding the impact of NPs exposure on the nutritional value of microalgae as food matrices. This includes examining the variations that trigger changes in the bioavailability and absorption of derived nutrients and on the synthesis of bioactive compounds (e.g., phenols) after a digestive process. Addressing these factors comprehensively is essential for ensuring the quality and safety of using plastics as contact materials for microalgae as food supplements.

## Supporting Information Available

Mass based size distribution of synthesized PE NPs using analytical ultracentrifugation and microalgae growth curves (PDF)

## Abbreviations

PS: polystyrene
PE: polyethylene
MPs: microplastics
NPs: nanoplastics
CV: *Chlorella vulgaris*
HP: *Haematococcus pluvialis*
PdI: polydispersity index
DLS: dynamic light scattering
TPC: total phenolic content
SOF: simulated oral fluids
SGF: simulated gastric fluids
SIF: simulated intestinal fluids
