# Supplementary material for: Impact of Nanoplastics on the Functional Profile of Microalgae Species Used as Food Supplements: Insights from Comparative In Vitro and Ex Vivo Digestion Studies
Source: J Agric Food Chem. 2024 Dec 24;73(1):798–810. doi: 10.1021/acs.jafc.4c07368 (PMC11726606; doi:10.1021/acs.jafc.4c07368)

Supplementary material

Impact of Nanoplastics on the Functional Profile of Microalgae Species Used as Food Supplements: Insights from Comparative In Vitro and Ex Vivo Digestion Studies

Davide Lanzoni[a+], Marisa *Sárria Pereira de Passos*[b+†], *Dora Mehn*[b], *Sabrina Gioria*[b*], António A. Vicente[c], *Carlotta Giromini*[a,d].

[a] *Department of Veterinary and Animal Science (DIVAS), Università degli Studi di Milano, Via dell'Università 6, 29600 Lodi, Italy.*

[b] *European Commission, Joint Research Centre (JRC), 20127 Ispra, Italy.*

[c] *CEB – Centre of Biological Engineering, University of Minho, 4710-057, Braga, Portugal.*

[d] *Institute for Food, Nutrition and Health, University of Reading, Reading, RG6 5EU, UK.*

[+]These authors contributed equally to this work.

[†] *Current address: Institute of Biotechnology, RWTH Aachen University, Worringerweg 3, 52074 Aachen, Germany.*

*Corresponding author: Sabrina Gioria. Email: sabrina.gioria@ec.europa.eu; Tel : +39 0332 783584

Characterization of size distribution and particles density of synthesized polyethylene (PE) nanoplastics (NPs) using analytical ultracentrifugation (AUC)

A centrifugal sedimentation method - analytical ultracentrifugation (AUC) - was used to characterize the size distribution and density of the *in-house* synthesized PE NPs. It measures the velocity at which particles move through a liquid medium under centrifugal force. Sedimentation velocity experiments can provide data on polydispersity and the presence of different size populations of NPs. The sedimentation coefficients depend on the particle size and density. In case both of these parameters are unknown, multiple experiments at different liquid medium densities can be performed to determine both the effective density and size distribution of nanoparticles (ISO 18747-2_2019).

A Proteomelab XL-I analytical ultracentrifuge (Beckman Coulter) equipped with interference and absorbance optics was therefore used to register the sedimentation of the particles in water and in 10 % water 90 % deuterated water (D_2O) mixture at 1000 rpm rotation speed in two sector sapphire window cells using the interference optics. The sample sector was loaded with 390 µL 10x diluted sample in water or in D_2O, while the reference cell contained 400 µL of the corresponding liquid medium. Interference data were fit using the ls-g*(s) model of the software sedfit with a linear grid in the -20000-0 S range at 100 resolution for water and in the -30000-0 S range at 150 resolution for D_2O. The modes of the resulting sedimentation coefficient distributions were inserted as sedimentation speed values in the equation suggested by the multi velocity approach ISO standard (ISO 18747-2:2019). For 10 % water 90 % D_2O mixture, liquid medium density and viscosity were determined by linear interpolation and values of 1.095 g cm^{-3} and 1.226 mPas were respectively applied. Particle density determined in this way (0.882 g cm^{-3}) was applied to transform the sedimentation coefficient distribution obtained in water to the particle size distribution shown in Figure S1. Considering that the interference signal is directly proportional to the mass concentration in a wide particle size range, the distribution generated can be considered as a mass based differential size distribution.

Figure S1

Mass based size distribution of synthesized PE NPs using AUC.

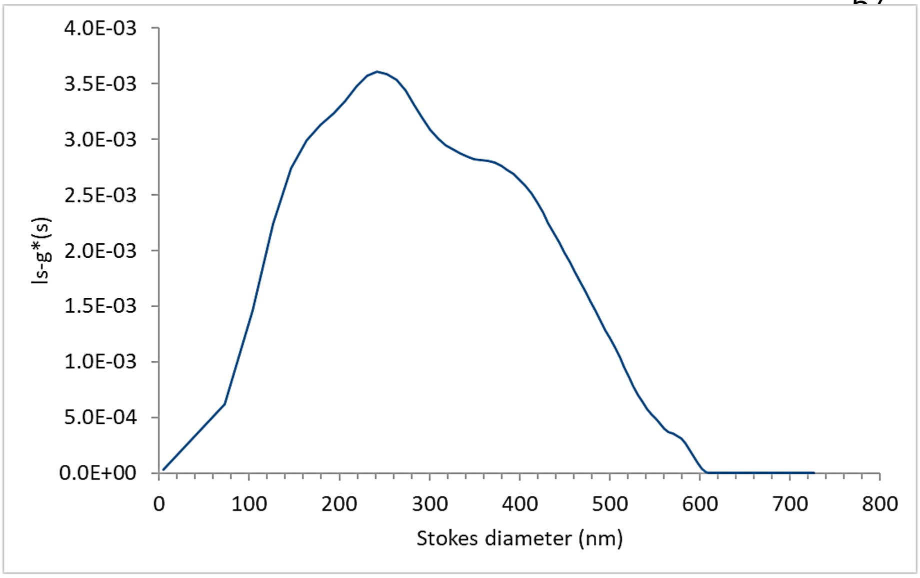

Figure S2

Microalgae growth curves: the number of cells mL^{-1} (cell density) was monitored in the corresponding growth media overtime by regular sampling and counting of the cells using a Neubauer chamber, under an ECLIPSE Ts2 inverted microscope coupled to a DS-Fi3 digital camera.

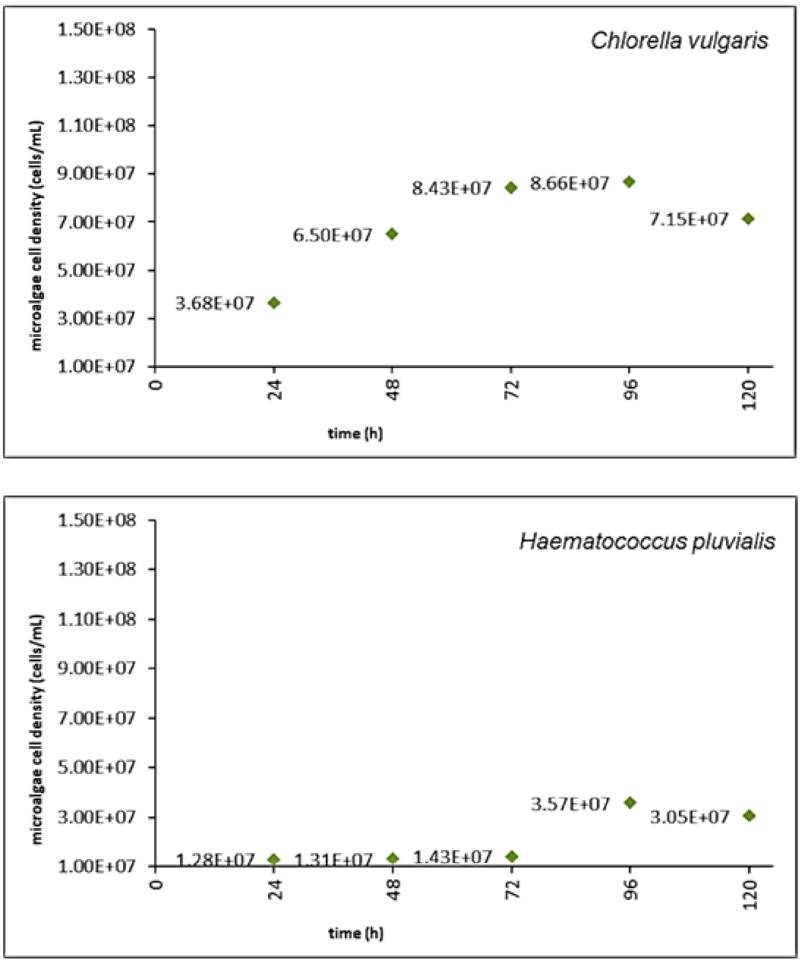

Supplement: Supplementary file 1 — jf4c07368_si_001.pdf [file jf4c07368_si_001.pdf]
